# Benzo  pyrene-induced DNA adducts and gene expression profiles in target and non-target organs for carcinogenesis in mice

**DOI:** 10.1186/1471-2164-15-880

**Published:** 2014-10-08

**Authors:** Jie Zuo, Daniel S Brewer, Volker M Arlt, Colin S Cooper, David H Phillips

**Affiliations:** Weatherall Institute of Molecular Medicine, John Radcliffe Hospital, Oxford University, Oxford, OX3 9DS UK; School of Biological Sciences, University of East Anglia, Norwich Research Park, Norwich, NR4 7TJ UK; Analytical and Environmental Sciences Division, MRC-PHE Centre for Environment & Health, King’s College London, Franklin-Wilkins Building, 150 Stamford Street, London, SE1 9NH UK; The Medical School, University of East Anglia, Norwich Research Park, Norwich, NR4 7TJ UK

**Keywords:** Carcinogenicity, Organotropism, Benzo[*a*]pyrene, DNA adducts, Gene expression, Microarray, miRNA

## Abstract

**Background:**

Gene expression changes induced by carcinogens may identify differences in molecular function between target and non-target organs. Target organs for benzo[*a*]pyrene (BaP) carcinogenicity in mice (lung, spleen and forestomach) and three non-target organs (liver, colon and glandular stomach) were investigated for DNA adducts by ^32^P-postlabelling, for gene expression changes by cDNA microarray and for miRNA expression changes by miRNA microarray after exposure of animals to BaP.

**Results:**

BaP-DNA adduct formation occurred in all six organs at levels that did not distinguish between target and non-target. cDNA microarray analysis showed a variety of genes modulated significantly by BaP in the six organs and the overall gene expression patterns were tissue specific. Gene ontology analysis also revealed that BaP-induced bioactivities were tissue specific; eight genes (*Tubb5*, *Fos*, *Cdh1*, *Cyp1a1*, *Apc*, *Myc*, *Ctnnb1* and *Cav*) showed significant expression difference between three target and three non-target organs. Additionally, several gene expression changes, such as in *Trp53* activation and *Stat3* activity suggested some similarities in molecular mechanisms in two target organs (lung and spleen), which were not found in the other four organs. Changes in miRNA expression were generally tissue specific, involving, in total, 21/54 miRNAs significantly up- or down-regulated.

**Conclusions:**

Altogether, these findings showed that DNA adduct levels and early gene expression changes did not fully distinguish target from non-target organs. However, mechanisms related to early changes in p53, Stat3 and Wnt/β-catenin pathways may play roles in defining BaP organotropism.

**Electronic supplementary material:**

The online version of this article (doi:10.1186/1471-2164-15-880) contains supplementary material, which is available to authorized users.

## Background

DNA adduct formation is an early critical event in the process of carcinogenesis by which many chemical carcinogens exert their biological effects. With few exceptions, however, carcinogens form DNA adducts in many tissues, leading to the view that adducts are necessary, but not sufficient, for carcinogenesis. Thus, although there are linear relationships between dose, adduct levels in target organs and tumour incidence [1], it may be difficult to identify which organs are targets for carcinogenicity by an agent purely from a consideration of the levels and persistence of DNA damage. Several studies using transgenic mice demonstrate that there is also a lack of correlation between carcinogen-induced increases in mutation frequency and target organ specificity [2–4]. These findings suggest the importance of events subsequent to DNA damage and mutation in determining organotropism and inter-species differences of carcinogens.

There have been reports of early gene expression changes in animal tissues following exposure to carcinogens or toxins, but thus far most studies have been limited to examinations of one target tissue with, usually, one non-target tissue [5–9]. No study has yet attempted to compare several target and non-target tissues in order to define the critical events subsequent to DNA damage and mutation that may determine carcinogen organotropism.

In the present study we have tested the hypothesis that carcinogen-induced changes in gene expression or miRNA expression may distinguish target organs from non-target ones. Balb/c mice were treated with multiple carcinogenic doses of benzo[*a*]pyrene (BaP), after which DNA adduct levels and gene and miRNA expression changes were measured in three target organs (lung, spleen and forestomach) and three non-target organs (liver, colon and glandular stomach). We found some similar expression changes in at least some of the target organs, but that the majority of the early gene expression changes were tissue specific, as were some of the biological processes related to the modulated genes. Nevertheless, the results shed new light on the complexity of the mechanism of BaP carcinogenicity and identify some responses that may determine the carcinogen’s organotropism.

## Results

### DNA adducts

**Figure 1:**
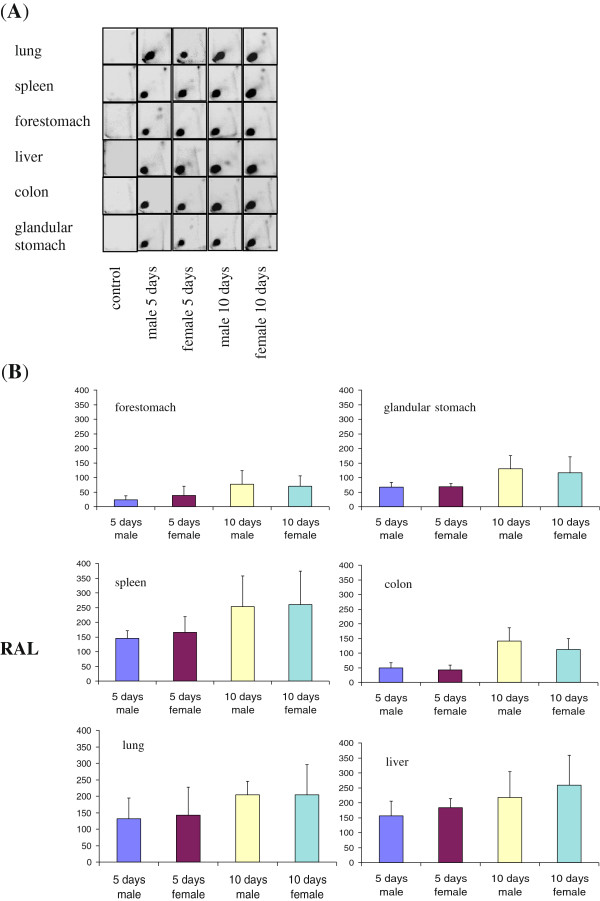
**BaP**–**DNA adduct formation in 6 Balb**/**c mouse organs.** The mice received 5 or 10 daily doses of 125 mg/kg b.wt/day BaP, or corn oil (control). **(A)** Autoradiographic profiles of DNA adducts obtained by TLC-^32^P-postlabelling. **(B)** Quantitative analysis of DNA adducts in mouse organs. Values represent the mean ± SD (n = 3). RAL; relative adduct labelling, adducts/10^8^ nucleotides. Differences between males and females were not significant (p > 0.05). Differences between 5 and 10 days were significant (p < 0.05) except for lung (p = 0.06).

^32^P-Postlabelling analysis of DNA from all six organs of BaP-treated mice (Experiment 1) showed a single adduct spot on TLC (Figure 1A). The radioactivity in this spot co-chromatographed with that produced by the standard benzo[*a*]pyrene diol-epoxide (BPDE)-modified DNA [10]. TLC plates of DNA from control animals were devoid of adducts (Figure 1A). Adduct levels in the six organs are shown in Figure 1B. For each organ, levels were not significantly different between males and females (Student’s *t*-test, *p* > 0.05). They were higher in mice treated with ten daily doses than in those treated with five: 50% higher in lung, 65% higher in spleen, 138% higher in forestomach, 41% higher in liver, 177% higher in colon and 80% higher in glandular stomach. The increases from five to ten doses were significant (*p* < 0.05) for all organs except lung. Differences in DNA adduct levels among the organs (1-way ANOVA) or between pairs of target and non-target organs (Tukey-Kramer test) were not significant (*p* > 0.05). Thus there was no significant distinction between target and non-target organs for DNA adduct formation.

### Gene expression profiles

By comparing pooled expression profiles from all BaP-treated samples from Experiment 2 (males and females at 3 different BaP doses) with corn oil-treated controls for each organ, we identified 791 genes in lung, 956 in spleen, 535 in forestomach, 309 in liver, 357 in colon and 939 in glandular stomach as significantly modulated by BaP (Welch’s *t*-test, *p* < 0.05), combining to a total of 2741 genes (Additional file 1: Table S1). This gene list was used for the subsequent cluster and functional analyses. Expression profiles were pooled in order to increase the power of the test and to reduce the weight of any one sample reducing the effect of outliers.

### Principal component analysis (PCA) and hierarchical cluster analysis (HCA)

**Figure 2:**
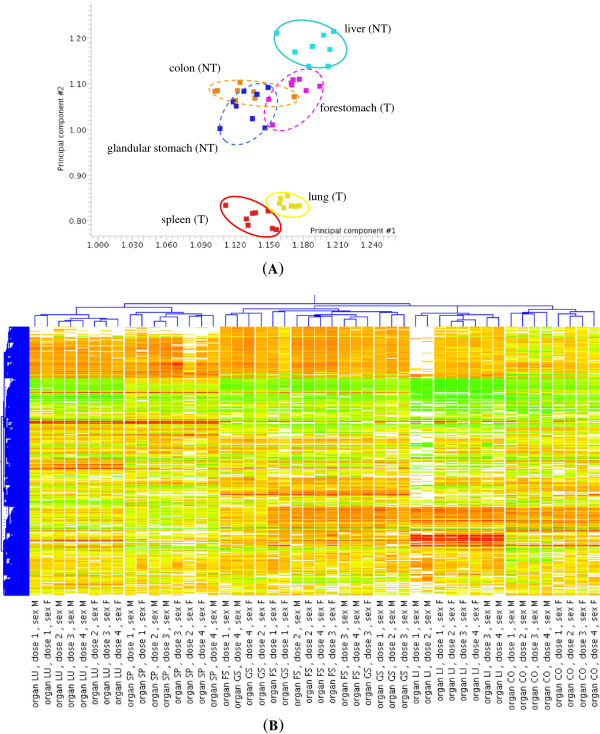
**Principal component analysis (PCA) and hierarchical cluster analysis (HCA). (A)** PCA of 2791 genes modulated significantly by BaP across 6 mouse organs**.** The first and second components were used (75.31% of total variances). The 8 hybridisation conditions in each of the 6 organs were grouped by gender and BaP dose (males or females, BaP dose of 125, 250 or 375 mg/kg b.wt/day and corn oil). T indicates target organs and NT non-target organs. **(B)** HCA of 2741 genes modulated significantly by BaP across 6 mouse organs. The 48 hybridisation conditions were clustered unsupervised. The results show tissue-specific expression patterns, especially in 2 target organs (lung and spleen). dose 1-corn oil, dose 2-BaP 125 mg/kg b.wt/day, dose 3-BaP 250 mg/kg b.wt/day, dose 4-BaP 375 mg/kg b.wt/day. LU-lung, SP-spleen, FS-forestomach, LI-liver, CO-colon, GS-glandular stomach.

PCA was used to analyse the expression profile of genes modulated significantly by BaP in at least one organ (2741 unique genes). Separate or nearly separate clusters were observed for lung, spleen and liver (Figure 2A). In contrast, there was overlap for the conditions in forestomach, colon and glandular stomach. The expression profiles of modulated genes uniquely define two of the target organs, lung and spleen. It is clear that the variations due to tissue differences are greater than variations due to BaP treatment.

In hierarchical clustering, the six profiles (3 doses × 2 genders) for each organ clustered together (Figure 2B). There was a closer similarity between the expression patterns of forestomach (target) and glandular stomach (non-target) than between any of the other organs. Again, the target organs lung and spleen clustered separately from the other organs. The corn-oil control samples clustered with the BaP-treated samples from the same organ again indicating that differences in expression profiles between organs were greater that the change induced by BaP.

### BaP-modulated gene expression in individual conditions

We identified 958 genes in lung, 1085 in spleen, 645 in forestomach, 523 in liver, 406 in colon and 1104 in glandular stomach that were significantly differentially modulated between at least one of the six conditions (3 doses × 2 genders) and corn oil-treated control samples (Welch’s *t*-test; *p* < 0.05). This represented 2770 different genes in total, of which 1906 genes are also among the 2741 genes identified by the pooling profiles method. To identify a consistent response we also considered genes significantly altered in at least three out of six conditions, for each tissue. There were 85 in lung, 108 in spleen, 97 in forestomach, 34 in liver, 25 in colon and 128 in glandular stomach (Representative genes are shown in Table 1; see Additional file 1: Table S2 for the full list). Using these criteria, only one gene *Hspa8* was found to be up-regulated in all six organs. *Hba*-*a1* was down-regulated in five organs.

**Table 1:** **Genes significantly modulated by BaP in individual organs** (**Welch**’**s** ***t***-**test**, ***p*** < **0.05**) **for at least three conditions**

Probe	Symbol	Gene name	EntrezID	M1*	M2	M3	F1	F2	F3
**Lung** **(target)**									
H3018D10	*Actb*	Actin, beta, cytoplasmic	11461	**1.310**	**1.280**	**1.290**	1.020	1.010	1.080
H3089D06	***Cav***	Caveolae protein 1	12389	**2.350**	**2.270**	**2.260**	**1.500**	**1.730**	**1.660**
H3110F10	***Ephx1***	Epoxide hydrolase 1, microsomal	13849	1.424	**1.696**	**1.658**	**2.220**	**2.382**	**2.910**
H3114C04	*Giyd2*	Giy-yig domain containing 2	75764	**0.530**	**0.800**	**0.610**	0.430	0.410	0.600
H3125H11	*Hba*-*a1*	Hemoglobin alpha, adult chain 1	15122	**0.597**	0.687	**0.487**	**0.502**	**0.442**	**0.580**
H3117D02	*Hbb*-*y*	Hemoglobin y, beta-like embryonic chain	15135	**0.445**	0.732	0.356	0.369	**0.217**	**0.405**
H3139E01	***Hspa8***	Heat shock protein 8	15481	1.687	**2.068**	**1.994**	**2.060**	**1.954**	**1.625**
H3097B08	*Pdia3*	Protein disulfide isomerase associated 3	14827	**1.211**	1.283	**1.388**	**1.270**	1.126	1.232
H3126C02	*Rpl18*	Ribosomal protein l18	19899	**1.350**	**1.400**	**1.490**	1.040	1.010	0.980
H3112G04	*Rps5*	Ribosomal protein s5	20103	**1.310**	**1.290**	**1.350**	0.970	0.860	1.000
H3068A11	*Tmed5*	Transmembrane emp24 protein transport domain containing 5	73130	**1.270**	**1.310**	**1.330**	**1.320**	1.120	**1.270**
**Spleen** **(target)**									
H3077H03	***Ccng1***	Cyclin g1	12450	**1.342**	1.362	1.665	**1.359**		**1.564**
H3114C04	*Giyd2*	Giy-yig domain containing 2	75764	**0.443**	**0.393**	0.513	0.484	**0.203**	0.383
H3126A10	*Hba*-*a1*	Hemoglobin alpha, adult chain 1	15122	0.540	0.661	**0.581**	**0.358**	**0.327**	**0.335**
H3134E01	*Hbb*-*y*	Hemoglobin y, beta-like embryonic chain	15135	0.813	**0.742**	**0.495**	**0.542**	**0.334**	**0.564**
H3051C06	***Hspa5***	Heat shock 70kd protein 5	14828	1.061	**1.359**	1.433	1.047	**1.241**	**1.522**
H3139E01	***Hspa8***	Heat shock protein 8	15481	1.297	**1.540**	**1.567**	1.937	**1.784**	**1.705**
H3097B08	*Pdia3*	Protein disulfide isomerase associated 3	14827	1.126	1.224	**1.388**	**1.161**	1.204	**1.425**
H3130D03	*Pip5k1c*	Phosphatidylinositol-4-phosphate 5-kinase, type 1 gamma	18717	**0.330**	**0.340**	**0.420**	0.640	**0.470**	**0.530**
H3139D02	***Tubb5***	Tubulin, beta 5	22154	**0.649**	**0.650**	**0.716**	**0.744**	**0.619**	**0.725**
**Forestomach** **(target)**									
H3011E09	*Actb*	Actin, beta, cytoplasmic	11461	1.210	1.860	**1.930**	**1.670**	1.520	**1.950**
H3089D06	***Cav***	Caveolae protein 1	12389		**0.570**	**0.630**	**0.570**	0.910	0.700
H3126H01	***Hba*** **-** ***a1***	Hemoglobin alpha, adult chain 1	15122	**0.210**	**0.240**	**0.150**	0.510	0.470	0.300
H3051C06	***Hspa5***	Heat shock 70kd protein 5	14828	1.332	1.837	**2.287**	**1.716**	1.687	**1.803**
H3139E01	***Hspa8***	Heat shock protein 8	15481	1.570	**2.382**	**2.752**	1.674	**1.956**	**2.153**
H3130D03	*Pip5k1c*	Phosphatidylinositol-4-phosphate 5-kinase, type 1 gamma	18717	**0.520**	**0.550**	0.740	0.590	**0.560**	**0.790**
H3126C02	*Rpl18*	Ribosomal protein l18	19899	**1.510**	**1.370**	**1.640**	0.950	1.030	1.160
H3112G02	*Rps5*	Ribosomal protein s5	20103	**1.320**	**1.270**	**1.440**	1.040	1.050	**1.360**
H3080H08	*Tmed5*	Transmembrane emp24 protein transport domain containing 5	73130	0.660	**0.690**	**0.720**	**0.610**	**0.580**	**0.530**
H3018E11	***Tubb5***	Tubulin, beta 5	22154	**0.588**	**0.624**	**0.576**	1.117	1.115	1.098
**Liver** **(non**-**target)**									
H3133B06	***Ccnd2***	Cyclin d2	12444	1.757	2.639	1.519	**1.724**	**1.532**	**1.924**
H3125H11	*Hba*-*a1*	Hemoglobin alpha, adult chain 1	15122	**0.170**	**0.420**	**0.490**	0.490	0.280	0.290
H3139E01	***Hspa8***	Heat shock protein 8	15481	0.935	1.592	1.354	**1.870**	**2.606**	**2.456**
H3129E07	*Rpl17*	Ribosomal protein l17	319195	1.130	**1.320**	**1.200**	1.220	1.370	**2.050**
**Colon** **(non**-**target)**									
H3139E01	***Hspa8***	Heat shock protein 8	15481	**1.397**	1.493	**1.559**	1.240	**1.577**	**2.110**
H3139D02	***Tubb5***	Tubulin, beta 5	22154	**1.951**	**2.106**	**2.216**	**1.318**	1.717	1.749
**Glandular stomach** **(non**-**target)**									
H3089D06	***Cav***	Caveolae protein 1	12389				**2.140**	**1.530**	1.300
H3126H01	*Hba*-*a1*	Hemoglobin alpha, adult chain 1	15122	**0.610**	**0.340**	**0.310**	0.310	0.250	0.240
H3051C06	***Hspa5***	Heat shock 70kd protein 5	14828	**1.316**	**1.780**	1.294	**1.253**	**1.661**	1.165
H3029C06	***Hspa8***	Heat shock protein 8	15481	1.242	**1.303**	1.046	**1.568**	**1.480**	1.266
H3029F12	*Rpl17*	Ribosomal protein l17	319195	1.340	**1.350**	**1.020**	1.400	1.400	**2.130**

*Columns M1, M2, M3, F1, F2 and F3 show the relative fold-change values in 6 exposure conditions (M1-male 125, M2-male 250, M3-male 375, F1-female 125, F2-female 250 and F3-female 375 mg/kg b.wt/day. Expression ratios in bold text mean being significant (*p* < 0.05) by Welch’s *t*-test. The genes shown in bold text were subsequently validated by Taqman gene expression assay.

The dose and gender effects on gene expression were investigated with 2-way ANOVA for each of the six organs. We did not find any clear dose- or gender-dependent expression in any of the organs.

### Target *versus*non-target gene comparisons

When comparing the genes modulated by BaP at three or more exposure conditions, no gene was significantly modulated in all target organs but not in any non-target organ, or vice versa. *Actb*, *Pdia3*, *Rpl18*, *Rps5* and *Tmed5* were identified to be up-regulated by BaP in comparison with corn oil-treated controls in two out of three target organs and in none of the non-target organs, whilst three genes were found to be down-regulated (*Giyd2*, *Hbb*-*y*, *Pip5k1c*). *Rpl17* was found to be up-regulated in two out of three non-target organs and in none of the target organs. *Tubb5* was found to be down-regulated in spleen and forestomach (two target organs) and up-regulated in the colon (one of the non-target organs).

Examining the results of the differential analysis performed on samples pooled by organ it was found that 25 probes had a significant modulation caused by BaP in the same direction for either all target organs or all non-target organs (Table 2; Welch’s *t*-test, *p* < 0.05). Of these, genes *Actg1*, *Crk and Dnajc3*, and clones *H3098D07* and *H3068A11* were found to be up-regulated in all target organs and down-regulated or not modulated in all non-target organs. *Psmd2* was down-regulated in target organs only and *Pfpl* was up-regulated in non-target organs only. *Sdccag1* was up-regulated in all organs and *Hspa5* in five organs. *Hbb*-*y* and *Hba*-*a1* were generally down-regulated in all organs.

**Table 2:** **Genes found to be significantly differentially expressed (Welch** ***t***-**test** ***p*** < **0.05) in the same direction for all target organs or all non**-**target organs**

					Average ratio of BaP-treated samples to oil treated control					
Clone ID	Symbol	Gene name	EntrezID	Genbank	LU	SP	FS	LI	CO	GS
H3098H09	2310016E02Rik	Riken cdna 2310016e02 gene	67695	BG071432	1.44	1.19	2.19	1.31	0.65	-
H3114E08	Actg1	Actin, gamma, cytoplasmic 1	11465	BG072752	1.20	1.17	1.27	-	-	-
H3134D01	AI461788			BG074374	1.53	1.28	1.41	2.02	-	-
H3083A04	Crk	V-crk sarcoma virus CT10 oncogene homolog (avian)	12928	BG069956	1.19	1.11	1.24	-	-	-
H3094A04	Dnajc3	DnaJ (Hsp40) homolog, subfamily C, member 3	100037258	BG071035	1.20	1.26	1.42	-	-	0.90
H3134F05	Gstm1	Glutathione S-transferase, mu 1	14862	BG074397	2.29	1.43	1.46	2.26	-	-
H3125H07	Hba-a1	Hemoglobin alpha, adult chain 1	15122	BG073608	0.59	0.54	0.57	0.26	-	0.42
H3125H08	Hba-a1	Hemoglobin alpha, adult chain 1	15122	CK334953	0.58	0.70	-	0.32	0.95	0.50
H3125H11	Hba-a1	Hemoglobin alpha, adult chain 1	15122	BG073610	0.54	0.77	0.40	0.35	-	0.37
H3126G09	Hba-a1	Hemoglobin alpha, adult chain 1	15122	CK334957	0.55	0.42	0.33	0.35	-	0.35
H3126H01	Hba-a1	Hemoglobin alpha, adult chain 1	15122	BG073679	0.58	0.44	0.28	0.25	2.02	0.32
H3140G04	Hba-a1	Hemoglobin alpha, adult chain 1	15122	BG074904	0.52	0.73	0.49	0.29	-	0.49
H3117D02	Hbb-y	Hemoglobin Y, beta-like embryonic chain	15135	BG072990	0.39	-	0.45	0.23	0.95	0.44
H3134E01	Hbb-y	Hemoglobin Y, beta-like embryonic chain	15135	CK335036	0.51	0.56	0.43	0.31	1.19	0.43
H3051C06	Hspa5	Heat shock protein 5	14828	CK334553	1.37	1.26	1.76	-	1.02	1.40
H3012A07	Hspa8	Heat shock protein 8	15481	BG063725	1.78	1.33	1.73	1.69	-	-
H3119B04	Hspa8	Heat shock protein 8	15481	CK334899	1.79	1.36	1.62	1.46	0.93	-
H3139E01	Hspa8	Heat shock protein 8	15481	BG074803	1.89	1.61	2.04	1.70	0.75	-
H3058G09	Mod1	Malic enzyme 1, NADP (+)-dependent, cytosolic	17436	BG067842	1.74	1.37	1.39	1.57	-	1.26
H3012G01	Pfpl	Pore forming protein-like	56093	CK334280	-	-	-	1.21	1.08	1.21
H3032C09	Psmd2	Proteasome (prosome, macropain) 26S subunit, non-ATPase, 2	21762	BG065537	0.87	0.89	0.84	-	1.50	-
H3082D02	Sdccag1	Serologically defined colon cancer antigen 1	71055	BG070064	1.59	1.58	1.53	1.52	1.05	1.46
H3106H07	Slco2a1	Solute carrier organic anion transporter family, member 2a1	24059	BG072114	1.46	1.22	1.32	-	-	1.19
H3068A11	Tecpr1	Tectonin beta-propeller repeat containing 1	70381	BG068669	1.27	1.19	2.00	-	0.68	-
H3131D02	Tnk2	Tyrosine kinase, non-receptor, 2	51789	BG074141	-	0.85	-	0.78	0.71	0.56
H3098D07					1.12	1.10	1.31	-	-	-
H3114F08					1.11	1.29	1.10	-	-	1.24

In the analysis samples were grouped by organ. All three doses are included. Ratios are only displayed for those organs where a significant difference was found. *LU* = lung; *SP* = spleen; *FS* = forestomach; *LI* = liver; *CO* = colon; *GS* = glandular stomach.

### Functional analysis of the modulated genes

We applied various bioinformatics approaches using the BaP-induced genes (see Additional file 1: Table S1) to identify which biological activities are modulated in BaP tumourigenesis. Firstly, in gene ontology (GO) analysis, the comparison of biological processes revealed considerable variation between the six organs (Additional file 1: Table S3). A few biological processes were common to all organs, and most of these were related to basic physiological activities like metabolic (GO:0008152), biosynthetic (GO:0009058) and macromolecule biosynthetic (GO:0009059) processes. Conversely, a number of processes showed tissue specificity, especially some cancer-associated processes, for example, response to oxidative stress in lung (six associated genes: *Dhcr24*, *Gpx3*, *Sod1*, *H47*, *Mtf1* and *Prdx6*) and cell growth in glandular stomach (11 genes: *Ube2e3*, *Alox12*, *Bcar1*, *Lepre1*, *Socs7*, *Sfn*, *Shc1*, *Esr1*, *Ppp2ca*, *Crim1* and *Yeats4*). Comparisons between organs suggested that processes including translational initiation (GO:0006413), polyamine metabolic process (GO:0006595), protein-RNA complex assembly (GO:0022618) and regulation of protein metabolic process (GO:0051246) represented target organ-specific functions while gas transport (GO:0015669) was the only process common to all the non-target organs. As we were assessing the effects of BaP treatment we also examined functions known to be involved in response to other external stimuli, such as response to stress (GO:0006950), to DNA damage stimulus (GO:0006974) and to oxidative stress (GO:0006979). We found that these appeared to be different in different organs, but do not fully distinguish target from non-target organs (although most do occur in two target organs). Other interesting activities such as DNA packaging (GO:0006323), apoptosis (GO:0006915) and cell proliferation (GO:0008283) were connected to cell cycle regulatory activities. Interestingly, these biological processes are also mainly altered in two target organs (e.g. cell cycle and cell proliferation).

Secondly, approximately half of the BaP-modulated genes in each organ were associated with regulatory or metabolic pathways. Over 90% of the pathways were found in more than one organ but most were common to two or three organs only. A few pathways, including cell cycle (genes including *Cdkn2A*, *Ccng1*, *Pcna*), MAPK signalling, Wnt signalling (e.g. *Apc*, *Src*, *Ccnd2*, *Cdh1*) and Jak-SAT signalling (*Stat3*), were modulated in all six organs (Additional file 1: Table S4), although different genes were found to be modulated in each case. For example, only *Ccnd2* expression in the Wnt signalling pathway was altered in all six organs. No pathway activated by BaP distinguished all three target organs from all three non-target organs.

**Figure 3:**
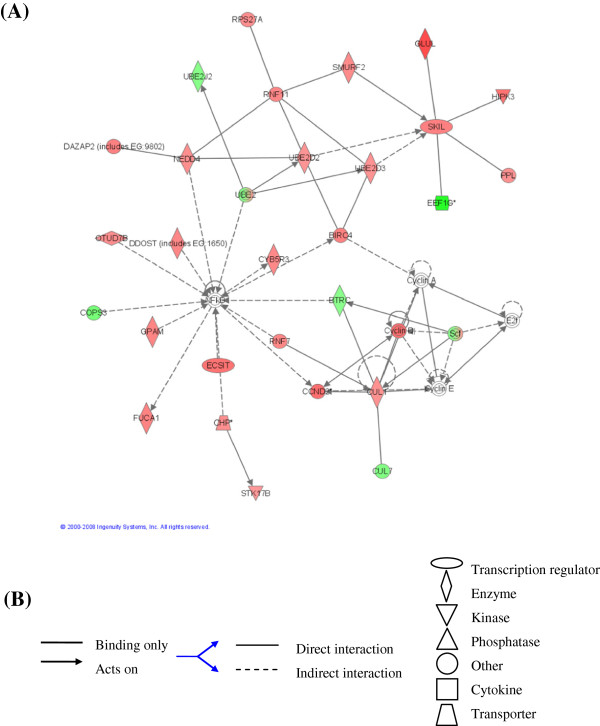
**Gene association network** (**GAN**) **3 in lung.** The network was generated with 791 BaP-significantly modulated genes by Ingenuity Pathway Analysis (IPA). **(A)** shows 28 BaP-modulated genes. The gene-gene interactions reveal that *NFκb* can be regulated by 10 genes (*Ecsit*, *Gpam*, *Ddost*, *Cops3*, *Birc4*, *Otud7b*, *Nedd4*, *Ube2*, *Btrc* and *Rnf7*) and it may regulate other 3 genes (*Fuca1*, *Cyb5r3* and *Ccnd2*). It also shows that core genes *NFκb* and *Ube* give central roles for the network functions protein synthesis and post-translational modification. Up-regulated genes are shown in red; down-regulated genes are shown in green. **(B)** the descriptions of interactions and transcriptional factors.

Thirdly, the interactive relationships among BaP-modulated genes in each organ were investigated by gene association network analysis using Ingenuity Pathway Analysis (IPA). Over 50% of the significantly modulated genes were present in networks. Networks commonly have one or more core (or ‘hub’) gene that links to many others within the network. *NFκb* in network 3 of lung (Figure 3), for example, can be regulated by 10 genes (*Ecsit*, *Gpam*, *Ddost*, *Cops3*, *Birc4*, *Otud7b*, *Nedd4*, *Ube2*, *Btrc* and *Rnf7*) and it may regulate three others (*Fuca1*, *Cyb5r3* and *Ccnd2*). Other core genes including *Erbb2*, *Ctnnb1*, *Fos*, *Myc*, *Trp53*, *TNF*, *Stat3*, *H*-*ras*, *Hspa8* and *Mapk* are associated with networks that had significantly modulated genes. Of these, *Fos* and *Hspa8* were themselves modulated. We did not find any network functions that distinguished the target from the non-target organs. Instead, cancer, cell cycle, protein synthesis and post-translation modification were network functions common to all six organs.

### Taqman gene expression validation

**Figure 4:**
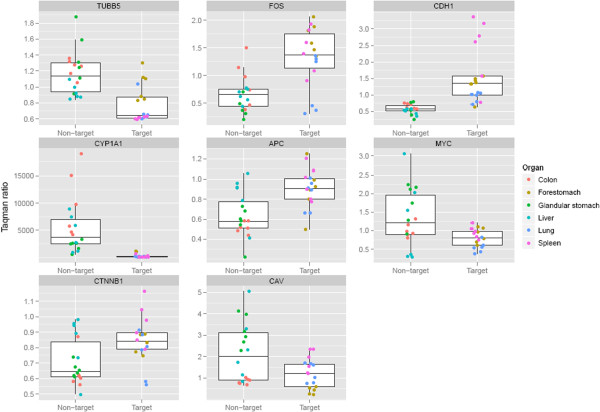
**Boxplots of the eight genes found to be modulated significantly differently between target and non**-**target organs** ***(p*** **<** **0.05;** **Benjamin**-**Hochberg multiple testing correction applied).** Expression values were normalised to the corn-oil control for each organ and pooled according to target or non-target status. Each organ is identified by colour. The box indicates the median, upper and lower quartiles and the whiskers are 1.5 times IQR (interquartile range; Q3-Q1) above/below the box.

The expression of 21 genes was measured by real-time PCR (RT-PCR). These were selected because they were differentially modulated by BaP between target and non-target organs, differentially modulated in most organs, core network genes or part of important pathways found to be modulated in the functional analyses. *Ephx1* [11, 12], *Cyp1a1* [13, 14] and *Trp53* [15] were chosen as they have previously been highlighted as important in BaP modulation. Table 3 shows the expression changes of these 21 genes, of which 11 genes modulated by BaP represent transcriptional differences between target organs and non-target organs *Trp53* and *Pcna* were down-regulated in two target organs (lung and spleen); *Cdh1*, *Ctnnb1* and *Apc* were down-regulated in non-target organs (*Ctnnb1 and Apc* in colon and glandular stomach, *Cdh1* in all three non-target organs); and *Src* was up-regulated in two target organs (lung and spleen) (Table 3; Full results in Additional file 1: Table S5). Additionally, *Ccng1*, *Ephx1*, *Cyp1a1*, *Stat3* and *Hspa8* were consistently up-regulated in the six organs. Looking at differences in expression between BaP-treated and control mice as a ratio and pooling according to target status, we found eight genes (*Tubb5*, *Fos*, *Cdh1*, *Cyp1a1*, *Apc*, *Myc*, *Ctnnb1* and *Cav*) to be modulated significantly differently between target and non-target tissue (*p* < 0.05; Figure 4). Even for these genes, a tissue might be more consistent with the opposite group rather than with its own. For example, for the top hit *Tubb5*, the gene with the most significant difference, forestomach seems to be more consistent with the non-target tissues.

**Table 3:** **Gene expression changes validation with RT**-**PCR**

Symbol	EntrezID	Gene name	LU	SP	FS	LI	CO	GS
**P53 related**								
*Trp53*	22059	Transformation related protein 53	↓	↓	F	F	F	F
*Pcna*	18538	Proliferating cell nuclear antigen	↓	↓	-	-	-	-
*Ccng1*	12450	Cyclin G1	↑	↑	↑	↑	↑	↑
**PAH metabolism**								
*Ephx1*	13849	Epoxide hydrolase 1, microsomal	↑	↑	-	↑	↑	↑
*Cyp1a1*	13076	Cytochrome P450, family 1, subfamily a, polypeptide 1	↑	↑	↑	↑	↑	↑
**Wnt/** **β**-**catenin signalling**								
*Cdh1*	12550	Cadherin 1	-	↑	-	↓	↓	↓
*Ctnnb1*	12387	catenin	-	-	-	-	↓	↓
*Apc*	11789	Adenomatosis polyposis coli	-	-	-	-	↓	↓
**Stat3 related**								
*Stat 3*	20848	Signal transducer and activator of transcription 3	-	↑	↑	↑	↑	↑
*Erbb2*	13866	v-erb-b2 erythroblastic leukemia viral oncogene homolog 2	↑	F	↓	F	↓	↓
*Src*	20779	Rous sarcoma oncogene	↑	↑	-	F	-	-
**Oncogene and tumour suppressor gene**								
*Fos*	14281	FBJ osteosarcoma oncogene	↓	↑	↑	↓	↓	↓
*Myc*	17869	Myelocytomatosis oncogene	↓	-	-	↑	-	↑
**Other genes**								
*Cav*	12389	Caveolin, caveolae protein 1	↑	↑	↓	↑	-	↑
*Tubb5*	22154	Tubulin, beta 5	↓	↓	-	-	-	-
*Cdkn2a*	12578	Cyclin-dependent kinase inhibitor 2A (P16)	-	↑	↑	↑	F	↑
*Ccnd2*	12444	Cyclin D2	-	↑	↓	↑	↑	-
*Hspa8*	15481	Heat shock 70kD protein 8	↑	↑	↑	↑	↑	↑
*Hspa5*	14828	Heat shock 70kD protein 5	↑	-	↑	-	↑	↑
*TNFα*	21926	Tumor necrosis factor alpha	↓	-	F	F	↑	F
*NFκb*	18033	Nuclear factor of kappa light chain gene enhancer in B-cells 1	↓	-	F	F	↓	F

The same RNA sample was measured 3 times independently for each exposure condition, and the consistent changes with a cut-off 1.5 occurring in 3 or more conditions in each organ was considered as being up- or down-regulated. ‘↑’ means up-regulation; ‘↓’ means down-regulation; ‘ – ’ means no change and ‘F’ means no data. *LU*, lung; *SP*, spleen, *FS*, forestomach; *LI*, liver; *CO*, colon; *GS*, glandular stomach.

### MicroRNA (miRNA) expression

**Figure 5:**
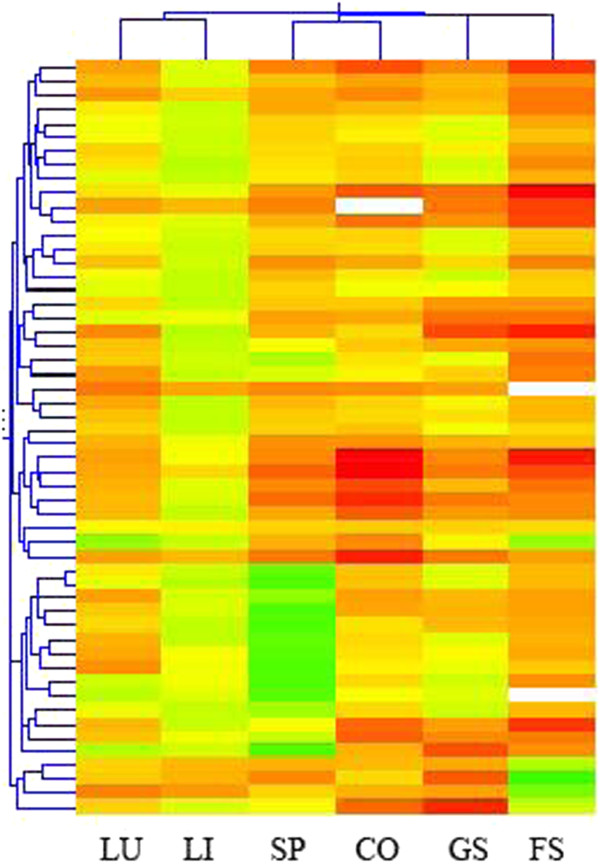
**Hierarchical cluster analysis** **(HCA)** **of 54 mouse miRNAs in 6 mouse organs.** The female mice received 5 daily doses of 125 mg/kg b.wt/day of BaP by oral gavage. 6 organs were unsupervised clustered with Pearson’s correlation analysis. The results show tissue specific expression patterns (LU-lung, LI-liver, SP-spleen, CO-colon, GS-glandular stomach, FS-forestomach).

The expressions of 54 mouse miRNAs on the microarray were first analysed using hierarchical cluster analysis (Figure 5). The majority of changes of miRNA expressions pattern were distinct for each of the six organs and the target organs did not cluster separately from the non-target organs. Instead, the results indicate that the profiles of BaP-induced miRNA expression changes in the six mouse organs are tissue specific.

**Figure 6:**
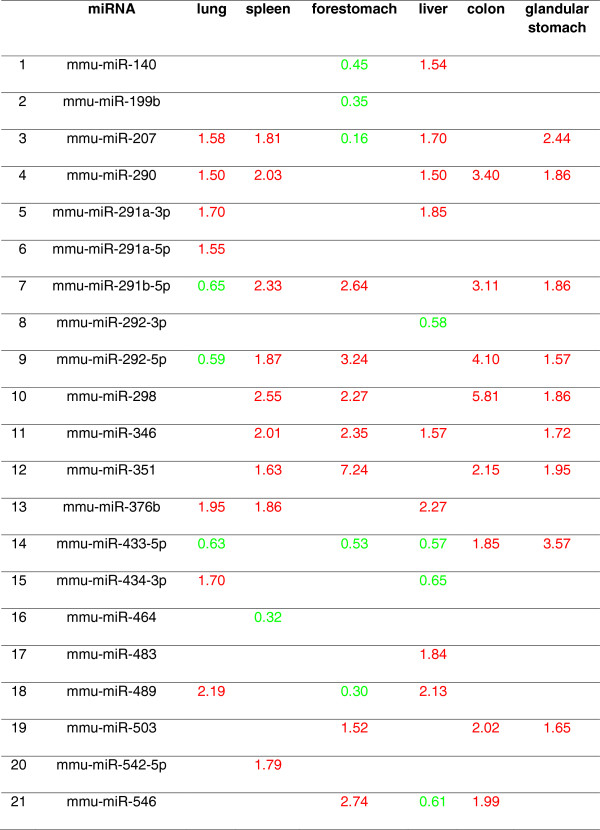
**The 21 miRNAs significantly modulated in 6 female mouse organs.** The mice received 5 daily doses of 125 mg/kg b.wt/day of BaP by oral gavage. The numbers represent fold change in expression relative to control organs (red, up-regulated; green, down-regulated). The deregulated miRNAs were filtered by relative expression ratios with fold-change cut-offs of 1.5 (*t*-test *p* < 0.05).

The relative miRNA expression values of the BaP-treated compared to control mice revealed the degree of modulation of miRNAs expression by BaP. With a cut-off of 1.5-fold, 41 out of 54 miRNAs displayed up-regulation or down-regulation in one or more organ (Additional file 1: Table S6). For instance, mmu-miR-290 was up-regulated in all organs except forestomach; mmu-miR-465 was down-regulated in lung, spleen and glandular stomach. Twenty one significantly deregulated miRNAs were identified in BaP-treated samples compared with control (*p* < 0.05, Student’s *t*-test; Figure 6). The number of miRNAs significantly altered in individual organs was as follows: 7 up, 3 down in lung; 9 up, 1 down in spleen; 7 up, 5 down in forestomach; 8 up, 4 down in liver; 8 up, 0 down in colon; and 9 up, 0 down in glandular stomach (Figure 6). These analyses did not identify any miRNA expression changes that were target organ-specific or non-target organ-specific.

## Discussion

BaP is a well-studied multi-organ and multi-species genotoxic carcinogen. Its carcinogenic potency and organotropism in mice depends both on the route of administration and on the dose [3, 16–19]. Hakura et al. [3] treated mice with 125 mg/kg b.wt/day of BaP by oral gavage for five consecutive days, which induced tumours in the forestomach, spleen and lung. In the present study, we treated Balb/c mice with BaP according to the same protocol. In addition, we administered this daily dose for a longer period (10 doses) in order to look for temporal changes in DNA adduct levels and gene expression. We also used 2- and 3-fold higher doses to investigate dose-related effects on gene expression. Forestomach, spleen and lung, the three target organs, were examined and we chose liver, colon and glandular stomach as non-target organs for comparison.

### DNA adduct formation

As expected, BaP induced the same DNA adduct pattern, consistent with metabolic activation to the 7,8-dihydrodiol 9,10-epoxide and covalent binding to the N^2^ position of guanine [20], in all organs examined. The similar levels of binding in males and females is consistent with the carcinogenic equipotency of BaP in both sexes, but the order of DNA adduct formation – spleen ≥ liver > lung > glandular stomach ≥ colon > forestomach – did not correlate with the susceptibility of the organs to BaP carcinogenicity [3].

Previous research has shown that mutation frequency in organs of BaP-treated mice was in the order of colon > forestomach > spleen > glandular stomach > liver = lung, while tumour incidence was in the order forestomach > spleen > lung after treating male MutaMouse with 125 mg/kg b.wt/day of BaP for 5 days by oral gavage [2, 21]. There are other examples of a lack of correlation between DNA adducts, mutation frequency and tumour incidence in different organs of carcinogen-treated mice [22, 23], although some level of correlation between mutation frequency and target organs [24–27], or between adduct levels and target organs [28], has been observed in some rodent studies.

While DNA adduct formation and mutation induction are important initiating events in carcinogenesis, these findings point to the possible role of subsequent events in determining the organotropism of chemical carcinogens. Thus in the present study we analysed microarray expression data from six organs of mice treated with BaP to look for tissue-specific changes. In total, data were collected from 144 hybridisations.

### Tissue-specific gene expression profiles

The analysis of the data from individual conditions (dose and gender) by PCA and by HCA showed that all datasets from a single organ usually grouped together.

Specificity of gene expression changes induced in tissues or cell lines has been reported previously. For example, brain, lung, liver and spleen each displayed a unique altered transcriptional signature in responding to infection [29]. Organ-specific gene expression profiles were found in C57BL/6 mice after low dose radiation, with the genes altered in spleen especially different from other organs [30]. We found that patterns from spleen and lung (target organs) were the most distinct. In human studies, Shyamsundar et al. [31] found tissue-specific expression patterns when comparing 35 human tumour tissues to their normal tissues. Other evidence for human tumour tissue gene expression specificity includes data presented for brain, breast, colon, endometrium, kidney, liver, lung, ovary, prostate, skin and thyroid cancers [32–34].

### Target organs *versus*non-target organs

Variations in the genes induced in target and non-target organs after carcinogen exposures have been reported, between kidney (target) and liver (non-target) following treatment of rats and mice with aristolochic acid [5, 9] or of rats with ochratoxin A [6], and between lung (target) and liver (non-target) of mice treated with BaP [7]. However, because only a single target and non-target organ were examined in these studies, it is possible that differences in the genes induced in the two tissues may represent organ-specific changes unrelated to carcinogenesis. The current study has addressed this limitation by comparing three target and three non-target organs.

Hundred of genes were significantly modulated by BaP in each organ and the PCA results revealed that the overall gene expression profile in the target organ group differed from those regulated in the non-target organ group. Interestingly, two target organs (lung and spleen) presented strong variations from the three non-target organs while another target organ, forestomach, was closer to the non-target organs. HCA confirmed this result. This finding is very close to another PCA result based on the expression data using oligonucleotide microarrays to capture splicing information of 22 mouse tissues [35], which showed clearly that the lung and spleen profiles were distinct from those of liver, colon and stomach. Additionally, Taqman results confirmed that several genes were modulated by BaP in a very similar pattern in lung and spleen, but different from the other four organs. p53 signalling pathway genes *Trp53* and *Pcna* were down-regulated, oncogene *Src* was up-regulated. *Tnfα* and *Nfκb* were down-regulated in lung and not changed in spleen. *Trp53* is one of the most studied tumour suppressor genes because its mutation or function loss has been reported in many human and animal cancers [36–38]. Recently a subset of genes were identified to show significantly different expression in p53 wild-type (WT) cells relative to p53-null cells after BPDE exposure in human colon carcinoma cells (HCT116), and the loss of this tumour suppressor influenced the metabolic activation of BaP [39].

However, forestomach (target) has an overall profile similar to that of the non-target organs, glandular stomach and colon. One possible reason is that the crucial gene expression changes in forestomach were very few constituting a low percentage of the total variances, for example, single gene deletion in forestomach tumourigenesis such as *Wwox* [40] and *c*-*H*-*Ras* [41]. Another possible reason is that the overall cell types between forestomach and glandular stomach are similar and the variations due to BaP treatment were not greater than the variations due to cell similarity.

Of the eight genes that were modulated significantly differently between target and non-target tissues (Figure 4), two are oncogenes (*Fos* and *Myc*) and three are involved in Wnt/β-catenin signalling (*Cdh1* – cadherin 1; *Ctnnb1* – catenin; *Apc* – adenomatous polyposis coli). *Cyp1a1* encodes the isoform of cytochrome P450 (CYP) that is principally responsible for BaP metabolism. *Tubb5* encodes class V Beta-tubulin, overexpression of which in mammalian cells disrupts microtubule organisation and spindle assembly, and blocks cell proliferation [42]. *Cav* encodes caveolae protein 1 (caveolin), a scaffolding protein that links integrin subunits to the tyrosine kinase FYN; it is a tumour suppressor gene candidate and a negative regulator of the Ras-p42/44 mitogen-activated kinase cascade. It can also facilitate the transit of Tyr-phosphorylated Stat3 into the nucleus [43] and integrin-mediated activation of PI3-K/Akt [44]. The over-expression of caveolin has been suggested as a biomarker in some human cancers [45, 46]. There is also evidence for the interaction of STAT3 with caveolin and HSP90 in plasma membrane raft and cytosolic complex [43]. Genes including Heat shock protein (Hsp) 70kD (Hspc8, Hspa5) and Hsp 90kD (Hspca) interact with Stat3 by binding to each other and positively activating each other; caveolin can assist in the binding procedure.

In non-target organs, Wnt/β-catenin signalling genes *Cdh1*, *Ctnnb1* and *Apc* were down-regulated in colon and glandular stomach. *Cdh1* was also down-regulated in liver and was the only gene that shows mRNA changes in all three non-target organs. E-cadherin is associated with invasion and metastasis of human cancers [47, 48] and can be regulated by the Wnt pathway. In unstimulated cells most β-catenin is associated with E-cadherin and α-catenin at the cell surface where it helps to mediate cell adhesion [49]. The Wnt/β-catenin signalling pathway can be regulated during carcinogenesis leading accumulation of β-catenin in the nucleus. This result is difficult to interpret in the context of carcinogenesis. The lack of change in expression of *Cdh1* in target organs would allow regulation to continue normally. However, since cell adhesion is decreased during carcinogenesis such down-regulation should not matter. But the story may not be this simple, because abnormal accumulation of β-catenin in both the cytoplasm and nuclei has been reported in during cancer development [50–53].

Although consistently up-regulated in five organs (not lung), *Stat3* was the only gene that exhibited a trend in dose-dependent changes in expression levels in the Taqman study based on RT-PCR analysis (Table 3). Dose-dependent changes in expression were observed in almost all exposure conditions in the organs. Stat3 is a member of a family of six different transcription factors that are closely linked with tumourigenesis. Activated Stat3 has been shown to protect tumour cells from apoptosis and promote cell proliferation by regulating genes coding anti-apoptotic and proliferation-associated proteins such as Bcl, Ccnd1 and c-Myc [54, 55]. Growing evidence suggests that Stat3 functions as an oncogene and plays a critical role in transformation and tumour progression. Stat3 may be activated by many growth factors and oncogenic kinases. These growth factors include EGF, TGF-α, IL-6 and hepatocyte growth factors. Oncogenic kinases include Janus kinase family (JAK) and Src [56]. Other reported factors include Cox2 [57], Erbb2 [58], JAKs & MAPK signalling [59, 60] and Itgb4 [61]. *Erbb2*, *Itgb4* and *Src* were differentially expressed in the 6 organs in this study.

### Functional analyses

cDNA microarray experiments identified hundreds of BaP-modulated genes in each of the six organs, and the potential significance of BaP-induced genes was investigated by the analyses of their involvement in particular biological processes, in regulatory or metabolic signalling pathways and in gene association networks.

GO analyses were consistent with the hypothesis that organs share some basic responses to BaP [62, 63]. Thus genes involved in basic physiological activities including cellular macromolecule metabolism and cellular physiological process were modulated in most or all organs.

Comparing the biological processes between target and non-target organs, it was found that very few changes were common in the target or in the non-target organs, examples being translation initiation and protein-RNA complex assembly. These processes may warrant further functional investigation, as many eukaryotic translation initiation factors were significantly modulated by BaP in lung (*Eif4g2*, *Eif3c*, *Eif4g3*, *Eif5 Mtif2* and *Eif1*), in spleen (*Eif5a*, *Eif5*, *Eif4g2*, *Eif4e* and *Eif1*) and in forestomach (*Eif3h*, *Eif5*, *Eif4e2*, *Mtif2* and *Eif4e*).

Processes identified mainly in target but also in non-target organs are those associated with cell cycle activity and cellular growth; or the response to external stimulus involving response to stress, response to heat and response to abiotic stimulus. Although these processes were mainly modulated in two target organs or in two non-target organs, individual cell cycle activities and responses to external stimulus are potential candidate categories for functions that might discern target organs from non-target organs. Under conditions of stress such as DNA damage, cell cycle controls many genes to carry out functions including DNA repair and apoptosis that repair the aberrant cells or cause cell death [64]. Carcinogens may induce tumourigenesis partly by mediating cell cycle malfunction. For instance, in MCF-7 cells BaP induces over-expression of many cell cycle genes including *TP53* and *CDKN1A* [39]; arsenate induces thymus atrophy mediated by cell cycle arrest by down-regulating E2F and related genes [65]; 2,3,7,8-tetrachlorodibenzo-p-dioxin (TCDD) makes cell cycle changes in murine fetal liver [66] and Rb loss abrogates cell cycle control and genome integrity to promote liver tumourigenesis [67]. The observation that many cell cycle genes are modulated significantly by BaP in two or more target organs indicates that processes such as DNA replication, cell cycle checkpoint and apoptosis may be activated to allow repair or elimination of damaged cells.

The biological processes response to stress (GO:0006950) and response to DNA damage stimulus (GO:0006974) were mainly found in the target organs. These processes are known to be linked to immune response [64] and it is well established that BaP modulates immune response; it suppresses the plaque-forming cell (PFC) response in adult male deer mice (*Peromyscus maniculatus*) [68]; it can accumulate the IgE antibody in serum and IL-4, IL-10, IL-12p70 and IFN-γ secreted by spleen cells in mice [69]; and it has been found be an immune suppressor at high doses, but an enhancer at low doses [70].

Over 90% of all pathways appeared in two or three organs and many of the individual pathways could potentially be linked to BaP carcinogenesis. It is also possible that pathways common to all or most organs are linked to tumourigenesis. MAPK signalling, for instance, is linked to BaP carcinogenicity: BaP may induce cell cycle arrest through activation of ERK/Cyclin D1 signalling [71]; BaP perturbs ERK/P38 pathway, which causes a decrease in stability and phosphorylation of p53 and down-regulation of NFκb activation [72]; and BaP activates MAPK signalling in mouse cl41 cells [73], in RAW 126.7 cells [74] and in A/J mice lung tumourigenesis [75]. Interestingly, the individual genes involved in MAPK signalling showed a strongly tissue-specific profile except for a very few common genes like *Hspa8*.

The Wnt signalling pathway was also commonly activated by BaP in all organs. The genes, including *Ccnd2*, *Csnk1a1*, *Ppp2r1a*, *Rock1*, *Cul1* and *Camk2b*, were variably modulated. In another study BaP caused LEF1 NH (2)-terminal mutation in K14DeltaNLef1 transgenic mice, which inhibits Wnt signalling and prevents induction of p53 in skin cancer [76]. In a study in which female mice were treated with 7,12-dimethylbenz[*a*]anthracene, Wnt signalling was up-regulated during mammary tumourigenesis [77].

The genes significantly modulated by BaP were analysed with IPA to show their regulatory relationships. Most network functions were related to cancer, cell cycle, protein synthesis and cellular development, which existed in almost all the organs. Interestingly some core genes showed organ-specific patterns, e.g. *Ap1*, *Sp1* and *PI3k*, but the majority of core genes were involved in all organs, including *Trp53*, *NFκ*b, *Tnfα*, *Fos*, *Stat3*, *Myc*, *Ifng* and *Erbb2*. If such pathways are of critical importance it is possible that the control of organ specificity of cancer development occurs at a different level with different pathways having different roles in different organs. For example *Cdkn2a* is generally involved in cell cycle control but can induce apoptosis in lung [78].

### miRNA expression

miRNAs are small non-coding RNAs of 20–22 nucleotides, typically excised from 60–110 nucleotide foldback RNA precursor structures [79–82]. They function at transcriptional or post-transcriptional levels through imperfect pairing with the target mRNA of protein-coding genes. miRNAs are related to crucial biological processes including development, differentiation, apoptosis and proliferation [80, 81]. Disregulation of miRNA function is linked to the development of a variety of cancers, including breast cancer [83, 84], B-cell leukaemia [85, 86], lung cancer [87, 88] and pancreatic cancer [89]. miRNA expression profiling appears in some instances to provide a better classification of cancer than conventional expression microarray studies [90, 91].

A variety of strategies may be used for miRNA detection, including a bead-based flow-cytometric approach [84], quantitative RT-PCR and serial analysis of gene expression (SAGE) [92]. In the present study oligonucleotide miRNA microarray analysis was used to assess miRNAs expression profiles. The purpose was to investigate whether alterations in miRNA profile following PAH exposure provides a better indicator of subsequent carcinogenic site-of-action than analysis of overall gene expression profile.

When the profiles of altered expression of 54 miRNAs for the 6 organs were analysed by hierarchical clustering, the profiles appeared to be tissue specific. Similar results have been reported in other studies [84, 92, 93]; for example, Liu et al. [94] reported that 245 miRNAs from humans and mice display tissue-specific expression signatures, and Babak et al. [91] found that more than half of 78 detected miRNAs were expressed specifically in 17 adult mouse tissues.

An interesting finding was that the magnitudes of the expression differences in the deregulated miRNAs were modest. The highest fold-changes were 5.8 for mmu-miR-298 in colon (up-regulation) and 0.164 for mmu-miR-207 in forestomach (down-regulation). 60% of the changes were smaller than 2-fold. A study in human breast cancer showed that over 90% of changes were 2-fold or less in 29 deregulated miRNAs [83]. Such small changes in human cancers are thought to be significant [85, 86]. The importance of small changes can be explained if individual miRNAs target many genes, and if the small changes can themselves significantly regulate individual target genes [92]. It has been shown that oncogenes and/or tumour suppressor genes may be disrupted dramatically by modulating miRNAs in human cancers; for example, miR-16-1 and miR-15a negatively regulate BCL2 production and miR-17-92 cooperates with c-Myc to stimulate proliferation [95].

Only two miRNAs were modulated by more than 2-fold in mouse liver in the present study (Figure 6). In another study, in which male B6C3F1 mice received 3 daily doses of 150 mg/kg b.wt BaP, the maximum fold change in a liver miRNA detected by microarray analysis was 1.35, but there were no changes in liver miRNAs that were statistically significant [96]. A similar study of miRNA expression changes induced in mouse liver by dibenz[*a*,*h*]anthracene found that most changes were small (<1.2 fold) and that only one miRNA that showed a dose-dependent increase [97]. Thus although miRNA response to environmental mutagens in liver may be a sensitive biomarker for liver carcinogens [98], it does not appear to be very sensitive for BaP, for which liver is a non-target organ in mice. Yauk and co-workers also found that in mouse lung 13 miRNAs were significantly modulated following 3 daily doses of 150 or 300 mg/kg b.wt BaP [99], but none of these were present in the microarray used in the present study (Figure 6).

The majority of miRNAs in this study were up-regulated by BaP. In this respect it is interesting that miRNA expression levels in tumour samples appears to be globally lower than in normal tissues. For example, 129 of 217 measured mammalian miRNAs were down-regulated in multiple human cancers [84]. Calin et al. [92] believe that these global miRNA levels facilitate the proliferation and transformation of cells via oncogenes genes such as *HRAS*. On the other hand, miRNAs can down-regulate gene expression during various critical cell processes including as apoptosis, differentiation and development [93] and negatively regulate target mRNAs [100]. The up-regulation of miRNAs in the 6 organs may also be associated with the down-regulation of target genes.

mmu-miR-292-5p is predicted to target *Erbb2* (miRBase database, http://www.mirbase.org/index.shtml). mmu-miR-292-5p was up-regulated in forestomach, colon and glandular stomach, and down-regulated in lung. This may explain the deregulation of *Erbb2* in these organs where Erbb2 was down-regulated in forestomach, colon and glandular stomach but up-regulated in lung (see *Erbb2* expression in Table 3). However, confirmation of the interactions of miRNAs and genes, such as mmu-miR-292-5p and *Erbb2*, needs further supporting evidence, e.g. confirmation of the modulation of mmu-miR-292-5p by BaP with RT-PCR, identification of the mechanism of mmu-miR-292-5p binding to *Erbb2*, or RNA interference to knock-out miRNA functions, as in the studies of miRNAs in carcinogenesis [101].

## Conclusions

In an investigation of the early changes induced in mouse organs exposed to a carcinogen, we determined whether DNA adduct formation, mRNA or miRNA expression can distinguish those mouse organs that are targets for BaP carcinogenesis (lung, spleen, forestomach) from three organs that are non-targets (liver, colon, glandular stomach). BaP-DNA adduct levels in mice did not distinguish target from non-target organs. miRNA expression patterns were modulated by BaP in a tissue-specific manner, and to a small extent. Gene expression profiles have provided a portrait of the overall tissue-specific expressions of six organs. Two target organs, lung and spleen, showed similar gene expression patterns which were different from the other four organs. p53 and Stat3 activities may suggest the mechanism that distinguishes these two organs. Conversely, there is little to distinguish the responses of the forestomach (target) from the glandular stomach (non-target). The modulation of *Cdh1*, *Ctnnb1* and *Apc* indicates Wnt/β-catenin activity specificity in non-target organs. Bioinformatic approaches suggest that cell cycle, immune response, MAPK signalling and Wnt signalling play critical roles in BaP carcinogenicity. Studies that describe carcinogen-induced gene expression differences between a single target organ and a single non-target organ should be treated with caution.

## Methods

### Animal treatment

Based on published protocols [4], the regimens for BaP treatment in this study are predicted to result in significant tumourigenesis in the long-term.

All animal procedures were carried out under licence from the UK Home Office in accordance with the Animals (Scientific Procedures) Act 1986, and with the approval of the Local Ethics Committee (Institute of Cancer Research).

In Experiment 1, groups of 5 male or female Balb/c mice (6 weeks old, ~25 g each) were administered daily doses of 125 mg/kg b.wt/day BaP (96% purity, Sigma-Aldrich, Poole, UK) (dissolved in 0.2 mL corn oil) by oral gavage. Some groups received 5 doses on days 1–5 and were killed on day 8. Others received a further 5 doses on days 8–12 and were killed on day 15. Control mice received corn oil only.

In Experiment 2, groups of 5 male or female Balb/c mice were administered 5 daily doses of 125, 250 or 375 mg/kg b.wt/day BaP on days 1–5 and killed on day 6. Control mice received corn oil only.

Six organs (lung, spleen, forestomach, liver, colon and glandular stomach) were collected from each mouse, snap-frozen in liquid nitrogen and stored at -80°C until analysis.

### ^32^P-Postlabelling analysis

Genomic DNA from the organs of 3 mice per treatment group in Experiment 1 was isolated by a standard phenol-chloroform extraction method and DNA adducts were measured using the nuclease P1 enrichment version of the ^32^P-postlabelling method as described [10]. Briefly, DNA (4 μg) was digested with micrococcal nuclease and calf spleen phosphodiesterase, then with nuclease P_1_ and ^32^P-labelled as reported. Solvents for 2-D thin-layer chromatography (TLC) of ^32^P-labelled adducts on polyethyleneimine-cellulose were as described [102]. TLC plates were scanned using a Packard Instant Imager (Dowers Grove, IL, USA) and DNA adduct levels were calculated from the adduct cpm and the specific activity of [γ-^32^P]ATP and expressed as adducts per 10^8^ nucleotides. An external BPDE-DNA standard [103] was employed for identification of adducts in experimental samples.

### cDNA microarray

Total RNA from the 6 organs treated in Experiment 2 was extracted by Trizol reagent (Invitrogen, Paisley, UK) and purified by Qiagen RNeasy mini kit (Qiagen, Crawley, UK) according to the manufacturer’s instructions. RNA was quantified spectrophotometrically, and integrity was determined using a 2100 BioAnalyzer (Agilent Technologies, South Queensferry, UK). RNA that had an rRNA 28S/18S ratio >1.2 was used.

Total RNA (4 μg) was reverse transcribed into cDNA and labelled with Cy5 dye (GE Healthcare, Chalfont St Giles, UK) using the Invitrogen indirect cDNA labelling kit protocol (Invitrogen) according to the manufacturer’s instructions. Four μg of universal mouse reference RNA (UMRR) (Stratagene, La Jolla, CA, USA) was labelled with Cy3 dye (GE Healthcare). After labelling, repetitive sequences within the cDNA samples were blocked with 10 μg mouse Cot-1 DNA (Invitrogen) to prevent non-specific sequences binding to the cDNA probes. Cy5-labelled test and Cy3-labelled reference were co-hybridised onto custom cDNA mouse microarrays containing 13,825 features (NIA 15 K Mouse cDNA clone set) [104]. Three total RNA samples from 3 mice were hybridised with UMRR. Slides were incubated at 42°C for 16 hours. Washes were performed once in 2× SSC and 0.1% (w/v) SDS for 15 min, followed by three washes in 0.1 × SSC and 0.1% (weight/vol) SDS for 10 min each, and two final washes in 0.1 × SSC for 2 min each, all at 65°C. Slides were spin dried before scanning on an Axon 4000A laser scanner (Axon Instruments, Union City, CA, USA) according to the manufacturer’s protocol.

### Microarray data preparation

The initial quality analysis of the scanned slides was carried out using GenePix Pro v-5.1 (Axon Instruments). A spot was automatically flagged for inclusion if the signal intensity of >75% of the pixels from either the Cy3 or Cy5 channels was 1 S.D. above background intensity. Spot quality was also assessed visually to fine tune the flagging. Raw data from GenePix were imported into GeneSpring v-7.2 (Agilent Technologies) without background subtraction and LOWESS normalisation [105] was applied. Natural log transformed averaged Cy5/Cy3 ratios were used in all subsequent analyses. PCA and HCA were performed on significantly modulated genes. The gene expression data discussed in this publication have been deposited in EMBL-EBI arrayExpress (https://www.ebi.ac.uk/arrayexpress/) with accession number E-MTAB-80.

### Pathway analysis

All genes modulated significantly by BaP in each organ were included for these analyses. Gene ontology (GO) [106] was performed by Database for Annotation, Visualization and Integrated Discovery (DAVID) [107]. In this study we concentrated on the significant (*p* < 0.05) biological processes. A second approach concentrated on gene interaction and cascade mechanisms like signalling transduction. The signalling annotation from pathway collections, including Biocarta, GenMAPP and KEGG were carried out using Pathway Miner [108]. Thirdly, gene association network (GAN) analysis, which examines all classes of relationships between genes, was carried out using Ingenuity pathway analysis (IPA) (Ingenuity systems, Redwood City, CA, USA).

### Taqman gene expression assay

cDNA was reversed transcribed from 1 μg total RNA (comprising equal amounts from 3 mice/group from Experiment 2) using random primers following the superscript III Reverse transcriptase first-strand cDNA synthesis protocol (Invitrogen). The cDNA was diluted 1:10 to 200 μL and 2 μL was used as template to perform Real-Time PCR in a 15-μl reaction. GAPDH was used as an endogenous control (Applied Biosystems, Foster City, CA, USA) in multiplexed PCR reactions on an ABI PRISM 7900HT Sequence Detection System (Applied Biosystems) with standard thermocycling conditions (50°C for 2 min, 95°C for 10 min, then 40 cycles at 95°C for 15 s, 60°C for 1 min), using Taqman Universal PCR Master Mix (Applied Biosystems). To confirm the modulated expression of the selected genes 20× Assays-On-Demand™ gene expression primers and probes (Applied Biosystems) were used.

The assay codes were: *GAPDH*-4352339E, *Trp53*-Mm00441964_g1, *Pcna*-Mm00448100_g1, *Ccng1*-Mm00438084_m1, *Ephx1*-Mm00468752_m1, *Cyp1a1*-Mm00487218_m1, *Cdh1*-Mm00486906_m1, *Ctnnb1*-Mm01350394_m1, *Apc*-Mm00545877_m1, *Stat3*-Mm00456961_m1, *Erbb2*-Mm00658541_m1, *Src*-Mm00436783_m1, *Hspa8*-Mm01731394_gH, *Hspa5*-Mm00517691_m1, *Fos*-Mm00487425_m1, *Myc*-Mm00487804_m1, *NFκB*-Mm00476361_m1, *Tnfα*-Mm00443258_m1, *Cav*-Mm00483057_m1, *Tubb5*-Mm00495804_m1, *Cdkn2a*-Mm00494449_m1, *Ccnd2*-Mm00438071_m1.

PCR reactions were performed in triplicate and changes in gene expression between the control (calibrator) and treated samples after normalisation to the GAPDH reference were calculated using the comparative threshold cycle (CT) method, where relative amount = 2^-ΔΔCT^ and ΔΔCT is the ΔCT of the target gene (threshold cycle test gene - threshold cycle endogenous control) minus the ΔCT of the calibrator sample (threshold cycle calibrator gene - threshold cycle endogenous control).

### MicroRNA

The 6 organs from 2 female BaP-treated (125 mg/kg/day for 5 days) and 2 corn oil-treated mice from Experiment 2 were used for analysis of miRNA expression. Supernatants containing total RNA, prepared as described above, were transferred to columns in miracle miRNA isolation kit (Stratagene, La Jolla, CA, USA) and miRNA isolated according to the manufacturer’s instructions. Samples were stored at -80°C until analysis.

The miRCURY LNA miRNA array (Exiqon, Vedback, Denmark; kindly provided by Dr Eduardo Missiaglia, Institute of Cancer Research; http://www.exiqon.com/array), which contains 54 mouse miRNAs, was used: 0.1 μg miRNA from BaP-treated mice was mixed with 8 μL 2.5× labelling buffer, 1.4 μL Cy5 fluorescent dye, 0.7 μL positive control, 14. μL labelling enzyme and the final volume adjusted to 20 μL with nuclease-free water. Another 0.1 μg miRNA from corn oil-treated mice was mixed with the same reagents, except for Cy3 instead of Cy5. Incubation was at 0°C for 1 hour, then at 65°C for 15 min to stop the labelling, after which the mixtures were concentrated to 7.5 μL and placed on ice.

The two mixtures were pooled and 5 μL 4× hybridisation buffer (GE Healthcare, Amersham, UK) was added. The solution was heated at 95°C for 3 min, centrifuged briefly, then applied to the arrays on a heating block at 63°C for 5 min, then covered with a glass cover slip and incubated at 63°C for 24 hours in a hybridisation chamber. Then the arrays were washed with solution A (20 mL 20x SSC, 4 mL 10% SDS, 176 mL HLPC-pure water) for less than 1 min at 60°C, followed by solution B (10 mL 20x SSC, 190 mL HPLC-pure water) twice for 2 min at room temperature, followed by solution C (2 mL 20x SSC, 198 mL HPLC-pure water) for 2 min at room temperature, and finally briefly with HPLC-pure water, then dried under a stream of air.

Scanning and image processing of the arrays was the same as for cDNA microarrays. The data were loaded into Genespring without background subtraction and normalised (per spot method), and modulated miRNAs identified by the fold-change method. Comparisons between the 6 organs were performed in Microsoft Office Excel. The miRNA expression data have been deposited in EMBL-EBI arrayExpress (https://www.ebi.ac.uk/arrayexpress/) with accession number E-MTAB-2407.

### Statistical analysis

For DNA adducts, comparisons between males and females and between 5-day and 10-day exposures were carried out using Student’s *t*-test (2-tailed). DNA adduct levels in 6 organs were tested by 1-way ANOVA and the Tukey-Kramer Test was used to identify significant differences (*p* < 0.05) between pairs of organs. For cDNA microarray data analyses, the significantly modulated genes were tested using Welch’s *t*-tests between test and control samples with test parameter non-adjusted *p* < 0.05. Two-way ANOVA was used to test for significant differences between males and females, as well as between the three levels of dosage with BaP. For HCA, Pearson’s correlation was used to calculate the distances with a Euclidean inter-group distance. For gene functional analysis, Fisher’s exact test was applied in gene ontology and pathway analyses. Pearson’s correlation was employed in analysing expression relationships between microarray and Taqman assay results. For RT-PCR data analyses, the differential expression was tested using Welch’s *t*-test with the Benjamin-Hochberg multiple testing correction. For the miRNA analyses, one-way ANOVA was used to test for significant differences between treated and control tissues.

### Availability of supporting data

The gene expression data have been deposited in EMBL-EBI arrayExpress (https://www.ebi.ac.uk/arrayexpress/) with accession number E-MTAB-80.

The miRNA expression data have been deposited in EMBL-EBI arrayExpress (https://www.ebi.ac.uk/arrayexpress/) with accession number E-MTAB-2407.

## Authors’ information

The authors were formerly in the Section of Molecular Carcinogenesis, Institute of Cancer Research, Brookes Lawley Building, Cotswold Road, Sutton SM2 5NG, UK, where the work described in this article was carried out.

## Electronic supplementary material

Additional file 1: **Supplementary Tables.** (XLS 1 MB)

## Abbreviations

BaP: Benzo[*a*]pyrene
BPDE: Benzo[*a*]pyrene diol-epoxide
ANOVA: Analysis of variance
PCA: Principal component analysis
HCA: Hierarchical cluster analysis
GAN: Gene association network
GO: Gene ontology
miRNA: microRNA
IPA: Ingenuity Pathway Analysis
IQR: Interquartile range.
